# Interplay between apoptosis and autophagy in colorectal cancer

**DOI:** 10.18632/oncotarget.18663

**Published:** 2017-06-27

**Authors:** Hao-Ran Qian, Zhao-Qi Shi, He-Pan Zhu, Li-Hu Gu, Xian-Fa Wang, Yi Yang

**Affiliations:** ^1^ Department of General Surgery, Institute of Minimally Invasive, Surgery of Zhejiang University, Sir Run Run Shaw Hospital, School of Medicine, Zhejiang University, Hangzhou 310016, Zhejiang, PR China; ^2^ Department of Pharmacology, Hangzhou Key Laboratory of Medical Neurobiology, School of Medicine, Hangzhou Normal University, Hangzhou 310036, Zhejiang, PR China

**Keywords:** colorectal cancer, autophagy, apoptosis, cross-talk

## Abstract

Autophagy and apoptosis are two pivotal mechanisms in mediating cell survival and death. Cross-talk of autophagy and apoptosis has been documented in the tumorigenesis and progression of cancer, while the interplay between the two pathways in colorectal cancer (CRC) has not yet been comprehensively summarized. In this study, we outlined the basis of apoptosis and autophagy machinery firstly, and then reviewed the recent evidence in cellular settings or animal studies regarding the interplay between them in CRC. In addition, several key factors that modulate the cross-talk between autophagy and apoptosis as well as its significance in clinical practice were discussed. Understanding of the interplay between the cell death mechanisms may benefit the translation of CRC treatment from basic research to clinical use.

## INTRODUCTION

Colorectal cancer (CRC) refers to the development of cancer in the colon and the rectum. It is considered as one of the most commonly diagnosed malignancies and a leading cause of cancer death worldwide. It is estimated that 1.4 million people were newly diagnosed, and this disease resulted in 693,900 deaths in 2012 [1].

Multimodality therapy, including surgical resection, radiotherapy and chemotherapy, is currently applied for the treatment of CRC [2]. However, the five-year survival is poor in the CRC patients at advanced stage and chemotherapy resistance remains an unsolved problem for the management of this disorder. Despite targeted therapy using antibodies against vascular endothelial growth factor (VEGF) and epidermal growth factor receptor (EGFR) has achieved a considerably improved prognosis, the median survival period of patients with advanced CRC who received chemotherapy is still short (< 3 years) [2]. Novel therapeutic approaches and strategies are urgently needed for improving therapy responses and overcoming cancer resistance.

Apoptosis, a tightly controlled pathway, mediates cell death under physiological and pathological conditions, and has been known as a therapeutic target for CRC [3]. Autophagy is a cellular defensive pathway under starvation or upon stimuli that regulates the survival and death of CRC cells [4]. Emerging evidence highlight the importance of the interaction between these two cellular processes in CRC cells undergoing physiopathological changes. In this review article, we focus on the recent findings about the interplay between the two life and death partners in CRC. Our study provides valuable evidence for the translation of autophagy- and apoptosis-based basic science into clinical practice.

### Outline of cell death machinery

#### The mechanism of apoptosis

Apoptosis is mainly divided into two signaling pathways, namely the intrinsic (mitochondrial mediated) and the extrinsic pathways. The intrinsic apoptosis initiates from intracellular stimuli, and is highly regulated by B-cell lymphoma-2 (Bcl-2) family members, such as pro-apoptotic proteins Bcl-2-associated X (Bax) and Bcl-2 homologous antagonist/killer (Bak) as well as anti-apoptotic members Bcl-2 and B-cell lymphoma-X large (Bcl-xL) [5]. The pro-apoptotic proteins trigger the mitochondria-mediated cytochrome C release and induce Caspase cascade activation which ultimately leads to cell death. Such process can be inhibited by anti-apoptotic proteins. Extrinsic pathway is initiated *via* the activation of the death receptors. Induction of apoptosis is accepted as the principle aim for most anti-cancer therapies, including chemotherapy, radiotherapy, and immunotherapy [6].

#### The mechanism of autophagy

Autophagy is an evolutionarily conserved intracellular degradative pathway which removes cytoplasmic organelles and long-lived proteins upon starvation and serves as a self-defensive process [7]. Three subtypes of autophagy have been characterized, including macroautophagy, microautophagy, and chaperone-mediated autophagy (CMA) [8]. Macroautophagy begins with the formation of phagophore, which engulfs cytosol and delivers for degradation in lysosomes [9]. Microautophagy is the process by which lysosomes govern the engulfment of cytoplasmic materials. CMA is an autophagic pathway mediated by molecular chaperones. In this study, we mainly discuss the involvement of macroautophagy (hereafter referred to autophagy) in determining the fate of CRC cells.

### Interplay between autophagy and apoptosis in CRC cells

#### Concomitant occurrence and independent regulation

**Figure 1:**
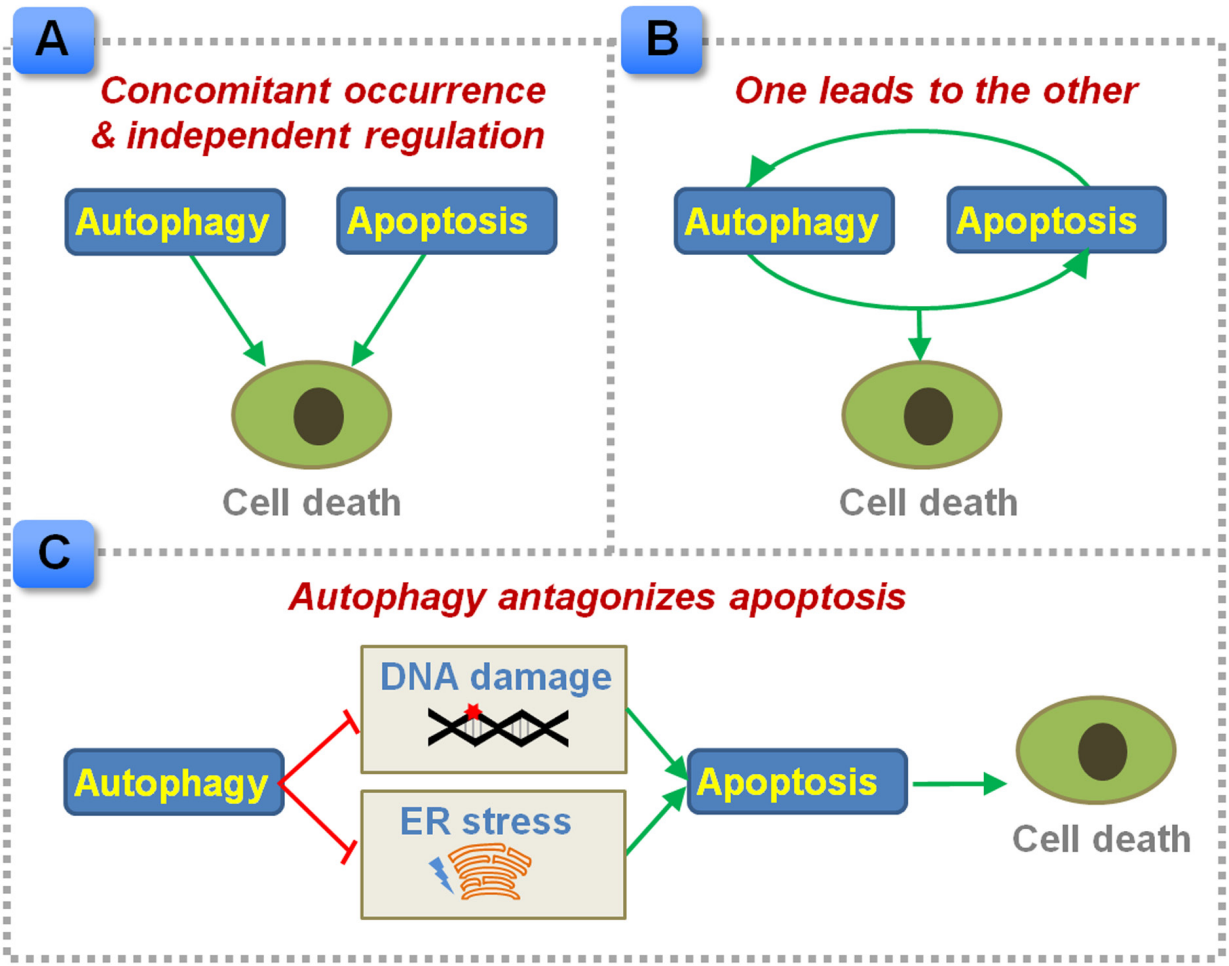
Proposed interplay between autophagy and apoptosis in regulating CRC cell death **(A)** Autophagy and apoptosis can be induced concomitantly, and regulate cell death independently. **(B)** Autophagy and apoptosis, one leads to the other. **(C)** Autophagy antagonizes apoptotic cell death by preventing the accumulation of damaged DNA and ER stress products.

Concurrent induction of apoptosis and autophagy has been documented in CRC cells and tumor-bearing nude mice in response to chemotherapeutic exposure or gene interference [10–15]. Autophagy may parallel with apoptosis in executing CRC cell death. Sequential occurrence of autophagy and apoptosis has been noted in CRC cells exposed to purvalanol, as purvalanol treatment induces an early autophagic response followed by apoptosis in HCT116 cells [16]. Moreover, these two programmed cell death pathways may drive cell death separately. Autophagy serves as an alternative cell death signaling in Bax and PUMA deficient human colon cancer cells that fail to undergo apoptosis [17]. Consistently, autophagy alone elicits cell death in apoptosis-resistant colon cancer cells [18]. On the other hand, apoptosis executes cell death in autophagy defective cells [19]. Based on this evidence, autophagy and apoptosis may act as cell death executioner, and drive cell death alternatively (Figure 1A).

#### One leads to the other

Inhibition of apoptosis using a pan-Caspase inhibitor z-VAD-fmk or by knocking down of Caspase-2 also blocks autophagy in HCT116 cells [20], indicating apoptosis may act as an upstream mediator of autophagy and activate autophagy in CRC cells (Figure 1B). In contrast, apoptosis inhibitor does not affect the justicidin A-induced autophagy induction in HT-29 cells, while blockage of autophagy by 3-methyladenine or silencing of *autophagy related 5* (*Atg5*) inhibits apoptosis in these CRC cells [21]. In this case, autophagy drives apoptosis instead of being a downstream effecter.

#### Autophagy antagonizes apoptosis

Mice deficient in *Atg5* exhibit increased expressions of cleaved Caspase 3 in colon tumors and reduced tumor size [22]. In accordance with this notion, pharmacological inhibition of autophagy or silencing of key autophagic genes has been shown to potentiate apoptosis signaling in CRC cells or colon cancer-bearing mice treated with anti-metabolites (e.g. 5-fluorouracil [5-FU] [23]), DNA disruption drugs (e.g. oxaliplatin [24, 25]), targeted therapy (e.g. celecoxib and an antagonist of Bcl-2/Bcl-xL [26], PI3K/mTOR inhibitor [27], BRAF inhibitor [28], niacin and tumor necrosis factor-related apoptosis-inducing ligand [29], store-operated Ca^2+^ entry inhibitor [30], SN-38/CPT-11 [31]), other compounds or extracts (e.g. icaritin [32], tetrandrine [33], apigenin [34], purvalanol [35]), radiation therapy [36] and photodynamic therapy [37]. Moreover, endoplasmic reticulum (ER) stress appears to be a crucial intermediate event as inhibition of ER stress has been found to suppress autophagy and subsequently enhance apoptosis in sodium butyrate-treated CRC cells [38]. Defective autophagy enhances DNA damage and apoptosis in CRC cells exposed to radiation [36]. In this case, autophagy plays a pro-survival role, which prevents the accumulation of DNA damage sensors and ER stress products in tumor cells and facilitates the evasion of apoptosis (Figure 1C). Blockage of autophagy might be used as a strategy to sensitize CRC to apoptotic cell death. Co-administration of a reagent that is capable of inhibiting autophagy with an apoptosis inducer, such as fluorouracil, may provide a promising way to improve the sensitivity to chemotherapy [39].

### Factors modulating the cross-talk between autophagy and apoptosis

Numerous factors and signaling pathways have been addressed to interconnect the autophagic and apoptotic pathways. Here, we discuss some of the apoptosis- or autophagy-related proteins that mediate the interplay between autophagy and apoptosis in CRCs (Table 1).

**Table 1:** Dural role mediators of autophagy and apoptosis and their resulting effects on CRC

Protein	Autophagy modulation	Apoptosis modulation	Expression in CRC	Role in CRC
**Apoptosis related proteins**				
Bcl-2	Binds to Beclin 1 and inhibits autophagy [43].	Anti-apoptosis [84]	Increased in low-grade and early-stage tumors [85].	Bcl-2 inhibition leads to the blockage of late stage autophagy in CRC cells [45]. The antisense oligonucleotide of Bcl-2 sensitizes Bcl-2-positive human colon cancer cells to radiation [40].
Bcl-xL	Binds to Beclin 1 and inhibits autophagy [86].	Anti-apoptosis [87]	Increased in CRC [41], and negatively associates with overall survival [88].	Oxaliplatin and bortezomib induce the dissociation of Bcl-xL from Beclin 1 and initiate autophagy in CRC cells [46]. Prevents CRC cell apoptosis, drives tumorigenesis and cancer progression [41].
Bax	Required for the autophagy regulation mediated by Bcl-2 and Bcl-xL [44].	Pro-apoptosis [89]	Increased in CRC [90], and decreases with cancer progression [91].	Restored Bax function by andrographolide promotes mitochondrial apoptosis and reverses 5-FU resistance [42].
p53	Mutant p53 inhibits autophagy [49].	Wild type p53, pro-apoptosis [48]; Mutant p53, anti-apoptosis [49].	p53 mutation occurs in 40%-50% CRC patients [48].	The p53 reactivating drug RITA induces DNA damage and sensitizes cells to 5-FU and oxaliplatin treatment [50].
**Autophagy related proteins**				
mTOR	Inhibits autophagy [51].	Shares common signaling in regulating apoptosis [52].	Increased in CRC [56]. Predicts poor prognosis for stage II CRC [57].	*Celastrus orbiculatus* extract induces the activation of autophagy and apoptosis in CRC cells through inhibiting mTOR signaling [54].
Atg5	Required for autophagosome formation [63].	Calpain-induced Atg5 cleavage induces apoptosis [58].	Decreased in CRC [59]. Absent expression of Atg5 associates with poor prognosis [60].	Knocking down of Atg5 sensitizes cells to apoptosis induced by icaritin [32] or propionate [61]. Heterozygous deletion of Atg5 promotes the anti-tumor efficacy of IFN-γ against intestinal adenomas in mice [62].
Beclin 1	Required for autophagosome formation [63].	Beclin 1 cleavage augments apoptosis [64].	Increased in CRC [66, 67]. High Beclin 1 expression associates with poor prognosis [68]. High Beclin 1 expression predicts better prognosis [69].	Knocking down of Beclin 1 sensitizes cells to icaritin-induced apoptosis [32]. Spicatoside A triggers the autophagy-to-apoptosis switch by inducing Beclin 1 cleavage [65].

5-FU, 5-fluorouracil; Bax, Bcl-2-associated X; Bcl-2, B-cell lymphoma-2; Bcl-xL, B-cell lymphoma-X large; CRC, colorectal cancer; IFN-γ, interferon-γ; mTOR, mammalian target of rapamycin; RITA, reactivating p53 and inducing tumor apoptosis.

### Apoptosis-related proteins

#### Bcl-2 family proteins

The expression of Bcl-2 family proteins is associated with the carcinogenesis and development of CRC. Inhibition of the anti-apoptotic proteins favors tumor growth suppression, as Bcl-2 inhibition sensitizes colon cancer cells to death [40], and mice deficient in Bcl-xL have reduced intestinal tumor burden [41]. Restored Bax function augments mitochondrial apoptosis and reverses 5-FU resistance in CRC cells [42]. In addition, close association between Bcl-2 family members and autophagy has also been noted. Bcl-2 and Bcl-xL bind to the BH3 domain of Beclin 1 and inhibit autophagy [43]. Such autophagy regulation governed by pro-survival Bcl-2 members requires the involvement of Bax and Bak [44]. Pharmacological inhibition of Bcl-2 hinders the Bcl-2-Beclin 1 interaction, and results in the blockage of late stage autophagy in CRC cells [45]. In contrast, the dissociation of Bcl-xL from Beclin 1 facilitates the Beclin 1-dependent autophagy induction in CRC cells [46].

#### p53

p53 is a tumor suppressor gene that functions in inhibiting tumorigenesis by inducing cell cycle arrest and apoptosis [47]. Mutation of p53, leading to the dysfunction of p53 pathway, occurs in around 40%-50% patients with CRC [48]. Mutant p53 gains of autophagy inhibition function through interacting with a variety of autophagy-related proteins [49]. Reactivating p53 induces DNA damage and improves cell responses to 5-FU and oxaliplatin [50].

### Autophagy-related proteins

#### mTOR

Mammalian target of rapamycin (mTOR) inhibits autophagy activation under basal conditions and serves as a negative regulator of autophagy machinery [51]. mTOR signaling pathway is regulated by diverse upstream signals, including AMP-activated kinase (AMPK) and phosphatidylinositol 3-kinase (PI3K) / protein kinase B (Akt) [52], both of which also contribute to apoptosis signaling in CRC cells in response to stimuli. For instance, activation of AMPK causes Caspase 8-mediated Beclin 1 cleavage and thus triggers apoptosis in CRC cells [53]. Inhibition of PI3K/Akt signaling pathway enhances CRC apoptosis [54, 55]. *Celastrus orbiculatus* extract induces the activation of autophagy and apoptosis in CRC cells through inhibiting mTOR signaling [54]. The protein expression of mTOR is higher in CRC than that in adjacent tissues [56], and the level of mTOR predicts poor prognosis for stage II CRC [57].

#### Atg5

Atg5 is an autophagy related gene participating in autophagy initiation. Yousefi et al. reveals an autophagy-to-apoptosis switch pattern induced by Calpain-mediated Atg5 cleavage [58]. After cleavage, Atg5 translocates to mitochondria and elicits cytochrome C release and mitochondrial apoptosis [58]. The protein expression of Atg5 is decreased in CRC [59], and absent expression of Atg5 has been implicated to associate with poor prognosis of CRC patients [60]. *In vitro* and *in vivo* studies demonstrate that Atg5 deficiency sensitizes CRC cells to apoptosis [32, 61, 62].

#### Beclin 1

Beclin 1, a mammalian ortholog of yeast Atg6, mediates the formation of autophagosomes and therefore contributes to autophagy initiation [63]. Caspase-modulated Beclin 1 cleavage results in the loss of its autophagy activation function, and the cleaved Beclin 1 augments apoptosis by promoting the release of pro-apoptotic factors from mitochondria [64]. In CRC cells, Beclin 1-dependent autophagy appears to be required for the maintenance of tumor cell survival, as knocking down of Beclin 1 sensitizes cells to icaritin-induced apoptosis [32]. Spicatoside A triggers the autophagy-to-apoptosis switch in CRC cells by inducing Beclin 1 cleavage [65]. Hence, Beclin 1 might be a critical mediator of the cross-talk between two cell death pathways. Compared to the weak expression of Beclin 1 in normal mucosal cells of colon, the level of Beclin 1 is higher in CRC tissues [66, 67]. The prognostic significance of Beclin 1 for CRC is still under debating. A meta-analysis enrolling six articles indicates that high Beclin 1 expression associates with poor prognosis of CRC patients [68]. However, another research group suggests that high Beclin 1 expression predicts better prognosis of CRC [69].

### Significance in clinical practice

Apoptotic signaling pathway is recognized as a potential therapeutic target for the management of CRC [3]. The efficacy of drugs targeting diverse mediators of the apoptotic cascade, such as death receptor 5 (DR5), Bcl-2 family proteins, and caspase activity, have been tested in pre-clinical and clinical trials for the treatment of CRC [70]. In recent years, great attention has been given to the importance of autophagic pathway in the chemotherapy resistance of cancer [71]. Autophagy is a novel and promising target for CRC therapy [4, 72] and may have considerable values for preventing the devastating occurrence of drug resistance. The mTOR inhibitors, temsirolimus [73] and everolimus [74], are rapamycin analogs that have been approved by the Food and Drug Administration (FDA) for the treatment of renal cell carcinoma. Phase I and phase II clinical trials of the autophagy stimulators for patients with CRC have been completed (For more details, See: www.clinicaltrials.gov). In a phase I clinical trial, a partial response is detected in one patient with advanced CRC after everolimus treatment [75]. A multicenter phase II study reveals that combined administration of everolimus with an angiogenesis inhibitor tivozanib achieves stable disease in 50% of patients with refractory metastatic CRC [76]. A recent study demonstrates that these rapamycin analogs are capable to provoke the extrinsic apoptotic pathway in CRC cells through regulating the DR5/Fas-associated protein with death domain (FADD)/Caspase-8 axis [77].

Autophagy inhibitor chloroquine (CQ) [78] and its analog hydroxychloroquine (HCQ) [79] impair autophagic degradation by disrupting lysosomal function. These agents have been approved by the FDA for the treatment of malaria, systemic lupus erythematosus, and rheumatoid arthritis. The anti-cancer activities of these autophagy inhibitors have been assessed in several clinical trials [80]. The efficacy of combined administration of HCQ and FOLFOX/Bevacizumab or Capecitabine/Oxaliplatin/Bevacizumab for CRC patients has been evaluated in phase I and phase II clinical trials [80]. In addition, a phase I clinical trial reveals that, combined use of HCQ with vorinostat, a histone deacetylase inhibitor, achieves stable disease in two patients with CRC [81]. Although the underlying mechanism remains largely unclear, the combined therapy with HCQ and vorinostat has been found to improve anti-tumor immunity in refractory metastatic CRC patients [82]. Nevertheless, further investigation with larger sample size of CRC is still required for examination of the treatment efficacy of HCQ. Based on the animal and cell culture studies as discussed above, combined use of autophagy modulator may sensitize the CRC cells to chemotherapy and overcome the chemotherapy resistance.

## CONCLUSION

Taken together, cell death signaling is complex and functions in coordination with one another. Here, we summarized the recent advances of the interplay between autophagy and apoptosis in modulating the survival and death of CRC cells. A better understanding of the molecular mechanisms involved in the carcinogenesis, progression and drug resistance of CRC may provide future directions of targeted therapies for this human malignancy. Nevertheless, it should be noted that, in addition to the classical programmed cell death processes mentioned in this study, other pathways, such as necrosis and necroptosis (programmed necrosis), also contribute to CRC cell death induced by anti-cancer therapy [70, 83]. Uncovering the mystery of cell death may open new window for CRC therapy. Moreover, there is considerable uncertainty regarding the prognostic values of the autophagy- or apoptosis-related factors in CRC. Evidence from large-scale clinical studies is therefore still required.
